# Generic switching: Do future physicians in Jordan have enough knowledge and a positive attitude?

**DOI:** 10.3389/fphar.2022.1037112

**Published:** 2022-12-06

**Authors:** Sura Al Zoubi, Lobna Gharaibeh, Batool Al-Masri, Ahmad B. Alsahele, Buthainah AL-Masaeid

**Affiliations:** ^1^ Department of Basic Medical Sciences, School of Medicine, Al-Balqa Applied University, As-Salt, Jordan; ^2^ Pharmacological and Diagnostic Research Center, Faculty of Pharmacy, Al-Ahliyya Amman University, Amman, Jordan; ^3^ School of Medicine, Al-Balqa Applied University, As-Salt, Jordan

**Keywords:** generic switching, generic substitution, pharmacists, medical student, knowledge, attitude, Jordan, drug policies

## Abstract

**Background:** Generic switching is a policy that has shown success in minimising pharmaceutical costs. It has also been used to mitigate recurrent and sudden drug shortages. Not all countries have policies that allow pharmacists to switch to generic drugs independently. In Jordan, only pharmacists at Ministry of Health hospitals automatically switch to generics if doctors had not already done INN prescribing.

**Objectives:** This study targeted medical students to assess their experience with generic switching as patients, their knowledge of the subject as students, and their attitude towards it as future prescribers and policymakers.

**Methods:** This is a descriptive, cross-sectional study conducted online. Eligibility criteria were being a fourth, fifth, or sixth-year medical school student enrolled at any of the six Jordanian universities. The questionnaire was developed by the researchers after a careful review of the relevant literature.

**Results:** Three hundred and ninety students responded to the online questionnaire. Most participants were females (244, 62.6%), senior students in their final (6th) year (162, 41.5%) and with very good academic achievement (166, 42.6%). The highest knowledge scores concerned patient rights (0.73/1.00), followed by knowledge about monitoring after generic switching (0.66/1.00), and patients with known drug allergies (0.66/1.00). Almost half of the participants believe that pharmacists should not be given the right to do generic switching and only 16% stated that they would choose generic drugs if they needed treatment in the future. Multivariate linear regression analysis showed that significant predictors of knowledge were gender, GPA, and family income. No correlations were found between participants’ knowledge scores and their attitudes towards giving pharmacists the right to independently switch drugs, or whether they would accept a substitute from pharmacists rather than having to refer to the physician.

**Conclusion:** Medical students in Jordan lack sufficient knowledge about generic switching. Students need to be more aware of the current policies and regulations of this practice, and the role of each healthcare worker involved in it. They also need to have a more positive attitude toward generic drugs and generic switching practice to facilitate its future implementation.

## Introduction

The huge cost of healthcare has become a global concern. Before the COVID-19 pandemic universal spending on healthcare was 10% of the gross domestic product (GDP) (World Health Orgaization, 2020). In Jordan, healthcare expenditure in 2019 was 7.58% of GDP, which is higher than most neighbouring or developing countries (The World Bank). Almost a quarter of Jordan’s healthcare expenditure is on pharmaceuticals, both over-the-counter (OTC) and prescription drugs (High Health Council, 2016). Compared to the Organisation for Economic Co-operation and Development (OECD) member countries, Jordan spends a higher proportion of its healthcare expenditure on drugs (International Federation pf Pharmaceutical Manufacturers, 2021). Many strategies have been tried by countries (including OECD countries), to reduce healthcare expenditure without jeopardising patient safety or treatment outcomes. One example of a policy that can result in significant reductions in pharmaceutical costs is generic switching (Moreno-Serra, 2013). This can result in major savings on pharmaceuticals, which accounts for the highest proportion of domestic out-of-pocket spending, making their cost catastrophic to some patients and households, especially in low and middle income countries (LMICs) (Cameron et al., 2009; Cameron et al., 2012; Ofori-Asenso and Adom Agyeman, 2016; Aregbeshola and Khan, 2018; Selvaraj et al., 2018).

Generic switching, or substitution, is when a branded drug that is prescribed by a physician is replaced, usually by the pharmacist, with a generic drug that is its bioequivalent and has similar efficacy, safety profile, active ingredients, dosage forms, routes of administration, and strength (Posner and Griffin, 2011; The Food and Drug Administration, 2021). Generic drugs only become available when the patent of the original branded drug expires and are usually much cheaper. Additionally, a single branded drug can have multiple generic alternatives, further reducing its cost due to the competition between manufacturers (The Food and Drug Administration, 2021). The price of some generic drugs may be as little as 2% of their branded equivalent at the time of patent expiry (Woerkom et al., 2012). Therefore, International Non-proprietary Name (INN) prescribing is supported by the World Health Organization (WHO) who consider it a rational drug use in both developed and developing countries (Almarsdóttir and Traulsen, 2005; Ofori-Asenso and Adom Agyeman, 2016). As a result, generic switching is a common practice in many countries, and even mandatory in others (Hassali et al., 2014), and can help prevent extravagant prescribing (Ofori-Asenso and Adom Agyeman, 2016). The use of generic drugs, however, faces the problem that some drug companies may falsely criticise their efficacy to protect the profits they make from selling the original branded drug (Baumgärtel et al., 2012; Generic bashing, 2013). In Jordan, pharmacists working in community pharmacies and the private sector are not allowed to independently do generic switching. Instead, they are legally obligated to refer to the prescriber before changing any drugs or making adjustments to prescriptions (Jordanian Ministry of Health, 2013; Alabbadi et al., 2014; Jordan Pharmacists Assocition, 2015). However, pharmacists at Ministry of Health hospitals are required to dispense the generic substitute if physicians did not do INN prescribing (Alabbadi et al., 2014).

Although cutting costs is the main reason for generic switching, other circumstances, such as drug shortages, have led community pharmacists in multiple countries to practice generic switching. Pharmacists in Australia reported using drug substitutions to overcome the drug shortages they have been facing since 2009 (Tan et al., 2016). In Canada, the drug shortage problem has been on the rise, and pharmacists are wasting large parts of their shifts dealing with this issue. Therefore, pharmacists used generic options to address the drug shortage issue (Panic et al., 2020). Aside from the common recurrent drug shortages, sudden and unexpected drug shortages can also occur due to political, natural, or medical crises. Recently, drug availability has been impacted in many countries due to COVID-19, and quick solutions were crucial to prevent any catastrophic consequences (Al Zoubi et al., 2021). Pharmacists played an important role at that time and tried several strategies, including generic switching, to mitigate the drug shortage problem (Badreldin and Atallah, 2021). The role of pharmacists was also widened in many countries to include generic switching in order to solve the drug shortages during COVID-19 (Merks et al., 2021). In the United Kingdom, despite the high INN prescribing rate and the few previous concerns about the dosage differences between some generics (Ferner et al., 2010), stakeholders appealed to England’s health and social care secretary to allow community pharmacists to make generic substitutions without referring to the general practitioners in case of drug shortages due to Brexit (Dyer, 2020).

Generic switching is considered safe for most drugs (Desai et al., 2019; Godman et al., 2021). Multiple research has shown that generic cardiovascular drugs (Manzoli et al., 2016), statins (Corrao et al., 2014; Gagne et al., 2014), antibiotics (Lin et al., 2016), and antihypertensives (Corrao et al., 2014) have comparable efficacy and safety to their originator drugs. Despite being an efficient way to reduce costs and overcome drug shortages, generic switching is not always appropriate. The safety and efficacy of some drugs with narrow therapeutic index (NTIs) is not guaranteed when switched (Al-Jazairi et al., 2008). The Food and Drug Administration (FDA) specify a group of NTI drugs that includes levothyroxine, immunosuppressants, antiepileptic drugs and antidepressants (Paveliu et al., 2011; Singh et al., 2014). Therefore, although currently less problematic, concerns remain about using or switching to generic immunosuppressants (Godman and Baumgärtel, 2015; Molnar et al., 2015). More research is needed before any clear recommendations about their safe substitution can be given. The case of antiepileptic drugs is both more complicated and more controversial. Studies show conflicting results, with many concluding that generic antiepileptic drugs are as safe and effective as the branded drugs (Kesselheim et al., 2010; Gagne et al., 2015; Lessing et al., 2015; Privitera et al., 2016; MHRA, 2017). In contrast, more recent advice from the Medicine and Healthcare products Regulatory Agency (MHRA), published on the UK government website, states that antiepileptic drugs can be assigned to 3 categories, with drugs from Category 1 (e.g., carbamazepine) showing clinical differences between generics and between generics and branded drugs. This suggests that generic switching should be avoided (Medicine and Healthcare products Regulatory Agency, 2017).

In some LMICs there are concerns about the quality of generics (Fadare et al., 2016). In Jordan, however, the practice of registering new generic drugs is strictly regulated and requires high-quality bioequivalence studies. There are laws such as the “Clinical Studies Law No. 2” and regulations such as the “Guideline for Assessing Bioavailability and Bioequivalence Studies,” all of which are published by the Jordan Food and Drug Administration (JFDA). This is in keeping with international guidelines such as the US FDA, the International Council for Harmonisation of Technical Requirements for Pharmaceuticals for Human Use (ICH) and The European Medicines Agency (EMA). Jordan’s advice concerning generics is continuously updated according to the most current of such international standards (The Jordanian Legislative Authority, 2011; Jordan Food and Drug Administration, 2017) and the country is considered a leader in Middle East research on bioequivalence and bioavailability (Shafout and Al Mahrouq, 2014; National Library of Medicine). There should, therefore, be no concern about the quality of generics in Jordan. Nevertheless, the global concern about generics belonging to specific drug classes (e.g., some NTI drugs) remains.

Accordingly, clear policies about generic switching, established by both pharmacists and physicians, should be implemented nationally, and internationally, to control and organise the practice to obtain the cost benefits without harming patients. Additionally, a list of switchable drugs should be made available for pharmacists, doctors, and other healthcare workers, to facilitate this practice. In Lebanon, for example, pharmacists were given the right to do generic substitution in 2010, and concurrently, the Ministry of Public Health published a substitution list on its webpage to act as a reliable source to which pharmacists should refer (Ministry of Public Health). Similarly in Norway, a medication exchange list was released by the Norwegian Medicines Agency in 2016 on their webpage for healthcare workers, and this list continues to be updated fortnightly (The Norwegian Medicines Agency, 2016). In Sweden, pharmacists have, since 2002, been obligated to switch the drugs that are listed on the Pharmaceutical Benefits Scheme to the cheapest available generic - unless that generic is not considered equivalent to the prescribed drug (Act, 2003; Andersson et al., 2005).

Consequently, physicians and pharmacists need to be familiar with currently applied policies and the details of the switching procedure. Many studies have highlighted the knowledge and attitude of patients, and the practice, knowledge, and attitude of pharmacists, pharmacy students, and physicians, towards generic switching (Colgan et al., 2015; Toverud et al., 2015). Such studies include those from Jordan (El-dahiyat and Kayyali, 2013a; El-dahiyat and Kayyali, 2013b; El-dahiyat et al., 2014), however to the best of our knowledge no research has been conducted in the country about generic switching from the perspective of medical students.

In this research, we targeted medical students to study their experience with generic switching as patients, their knowledge of the subject as current students, and their attitudes towards genetic switching as future prescribers and policymakers.

## Methods and materials

### Study design and population

This is a descriptive, cross-sectional study conducted online in the period from December 2021 to March 2022. The study’s questionnaire, design and protocol were approved by the Research Ethics Committee of the School of Medicine at Al-Balqa Applied University (BAU) (Reference Number: MD1305, Proposal Number: 21/2022). Respondents were medical students in their clinical training years. In Jordan, to obtain a Doctor of Medicine (MD) degree, students must complete a 6-year undergraduate medical program. In the first 3 years, students attend basic medical courses and in the remaining 3 years they attend clinical courses, do clinical training, start to interact with patients, and become familiar with drug use in clinical settings. Therefore, to be eligible to take part, participants had to be medical students enrolled at any of the six Jordanian universities that offer undergraduate medical courses, and in the 4th, 5th, or 6th year of their undergraduate studies. This ensured that they would be familiar with generic switching. A sample of 390 students was taken from the 6,192 students that meet these criteria. A minimum sample size requirement of 362 students was calculated for this study using Raosoft^®^ (Sample Size Calculator by Raosoft, 2022) (an online sampling calculator) to give a 5% margin of error and a confidence level of 95% and assuming a null response distribution of 50%. Participants were recruited using a convenience sampling method. The questionnaire was built using Google Forms and designed to be self-administered. Social media platforms were used to invite participants to fill out and submit the questionnaire anonymously and voluntarily.

### Questionnaire

The questionnaire was developed by the researchers in this study after a careful review of the relevant literature. The first version of the questionnaire was tested for face and content validity by a panel of experts from the field. After a pilot test was run, modifications were made where appropriate. Responses from the pilot run were excluded from the final analysis, and the final questionnaire was re-evaluated for validity. The questionnaire was written in English and contained 40 multiple-choice, close-ended questions. It was divided into four sections to collect information about participants’ demography, experience, knowledge, and attitude towards generic switching Supplementary Table S1. Before starting the questionnaire, there was a cover letter explaining the rationale of this study, outlining data protection and participant confidentiality measures, confirming the voluntary nature of participation and the participants’ right to terminate the questionnaire at any time and briefly defining some useful terms in the questionnaire such as brand drug, generic drugs, and generic switching in order to avoid any confusion or misunderstanding while filling the questionnaire.

### Statistical analysis

Data were analysed using IBM SPSS statistics (version 23) predictive analytics software. Categorical data were presented as frequencies and percentages and were compared using Pearson Chi-square (*χ* ^*2*^) test. Univariate and multivariate linear regression analyses were used to determine the predictors of knowledge score among participants. *p*-values of <0.05 were considered statistically significant.

## Results

### Participants’ characteristics

Three hundred and ninety (390) students from six universities across the country filled out and submitted the questionnaire. The majority of participants were females (244, 62.6%), come from families with income higher than the national average (278, 71.3%), have health insurance (318, 81.5%) and do not use drugs for the management of chronic diseases (341, 87.4%) but do regularly use drugs not related to chronic conditions (250, 64.1%) Table 1.

**TABLE 1:** General characteristics of the participants, *N* = 390.

	Frequency (%)
Gender	
Male	146 (37.4%)
Female	244 (62.6%)
Year of study	
Fourth-year students	109 (27.9%)
Fifth-year students	119 (30.5%)
Sixth-year students	162 (41.5%)
GPA	
Excellent (3.65–4.00)	76 (19.5%)
Very good (3.00–3.64)	166 (42.6%)
Good (2.50–2.99)	140 (35.9%)
Fair (2.00–2.49)	7 (1.8%)
Poor (<2.00)	1 (0.3%)
Family income is lower than the national average	
No	278 (71.3%)
Yes	112 (28.7%)
Do you own health insurance?	
No	72 (18.5%)
Yes	318 (81.5%)
Do you use any drugs that are not related to chronic medical conditions?	
No	140 (35.9%)
Yes	250 (64.1%)
Do you use any drugs for the management of chronic medical conditions?	
No	341 (87.4%)
Yes	49 (12.6%)

Participants were asked about their demographic information. Data are presented as frequency (percentage) [*N* (%)].

Participants were students from all clinical study years. However, most of them were senior students in their final (6th) year (162, 41.5%) and had received very good (GPA of 3.5–3.64) academic achievement (166, 42.6%) Table 1.

### Participant’s past experiences

Participants reported that from their previous experiences, doctors use specific brand/generic drug names in their prescriptions (184, 47.2%). Only 15.4% of the participants have never experienced any unavailability of drugs. In the cases where the drugs were not available (330, 84.6%), the majority of participants reported that the pharmacist changed the medication without referring to the prescriber. When participants were offered multiple substitutions, the majority (282, 72.3%) said that they had chosen the one they had used before and only (5, 1.3%) and (64, 16.4%) said that they had chosen the most expensive or the foreign drugs, respectively Table 2.

**TABLE 2:** Participant’s past experiences towards generic switching, *N* = 390.

	Frequency (%)
Prescriptions contained the active ingredient or the brand/generic name of the drug	
Active ingredient	27 (6.9%)
Brand/generic name	184 (47.2%)
Both	105 (26.9%)
I do not remember	74 (19.0%)
Availability of prescribed drugs in the pharmacy	
Drugs were always available	60 (15.4%)
Drugs were not available a few times	221 (56.7%)
Drugs were not available many times	109 (27.9%)
When the drug was not available (may choose more than one answer), *N* = 330	
The pharmacists asked me to look in another pharmacy	120 (36.4)
The pharmacist offered to order it for me	77 (23.3)
The pharmacist called the physician for a substitution	52 (15.8)
The pharmacist offered me a substitution without referring to the physician	234 (70.9)
Have you ever asked the pharmacist for a substitution for a prescribed drug?	
No	244 (62.6%)
Yes	146 (37.4%)
Have you ever switched from a certain brand to another brand or generic drug?	
No	176 (45.1%)
Yes	214 (54.9%)
When you were offered multiple generic drugs, which one did you choose? (you can choose more than one)	
The cheapest drug	93 (23.8%)
The most expensive	5 (1.3%)
The drug I used before	282 (72.3%)
Drugs manufactured by drug companies outside Jordan	64 (16.4%)
Drugs manufactured by drug companies that I trust	136 (34.9%)
Only the one recommended by the physician	3 (0.8%)
Have you noticed any change in drug availability due to COVID-19?	
No	90 (23.1%)
Yes	117 (30.0%)
I do not know	183 (46.9%)

Participants were asked about their experience with generic switching. Data are presented as frequency (percentage) [N (%)].

One hundred and seventeen (117) participants noticed a change in drug availability due to the COVID-19 pandemic Table 2.

### Participant’s knowledge

Participants were asked questions to measure their knowledge about patients’ rights, pharmacists’ rights, the safety of generic switching, differences between brand and generic drugs, monitoring after drug substitution and generic switching in special populations. The highest knowledge score was in the category concerning patients’ rights (0.73/1.00), followed by knowledge about monitoring after generic switching (0.66/1.00), patients with known drug allergies (0.66/1.00), differences between generic and brand drugs (0.61/1.00), pharmacists’ rights (0.56/1.00), and finally safety of drug switching of different pharmacologic categories (0.41/1.00), Table 3.

**TABLE 3:** Evaluation of knowledge on different aspects of the generic switching process, *N* = 390.

	Number (frequency %)		Average score
	Correct answer	Wrong answer	
Knowledge of patients’ rights (1 Point)			0.73
Patients have the right to ask for a change in prescribed drug	283 (72.6%)	107 (27.4)	
Knowledge of pharmacists’ rights (1 Point)			0.59
Pharmacists have the right to independently change a drug a doctor prescribed for a patient	232 (59.5%)	158 (40.5%)	
Knowledge concerning safety of drug switching (1 Point)			0.41
The following drugs can be substituted safely			
Non-opioid pain killers	300 (76.9%)	90 (23.1%)	
Neurological disorder drugs (e.g., anti-epileptic)	295 (75.6%)	95 (24.4%)	
Immunosuppressants	287 (73.6%)	103 (26.4%)	
Hormones (e.g., levothyroxine)	216 (55.4%)	174 (44.6%)	
Antihistamines	187 (47.9%)	203 (52.1%)	
Contraceptives	126 (32.3%)	164 (67.7%)	
Antibiotics	124 (31.8%)	266 (68.2%)	
Corticosteroids	94 (24.1%)	296 (75.9%)	
Inhalers	86 (22.1%)	304 (77.9%)	
Cardiovascular drugs (ACEIs, ARBs, BBs, CCBs)*	40 (10.3%)	350 (89.7%)	
Antiplatelet and anticoagulants	40 (10.3%)	350 (89.7%)	
Knowledge of differences between generic and brand drugs (1 Point)			0.61
Efficacy	241 (61.8%)	149 (38.2%)	
Safety	213 (54.6%)	177 (45.4%)	
Cost	258 (66.2%)	132 (33.9%)	
Knowledge of the need for monitoring after the drug switch (1 Point)			0.66
Generic switching in chronic diseases needs more frequent monitoring	259 (66.4%)	131 (33.6%)	
Knowledge of allergy and drug switch (1 Point)			0.66
People who have experienced an allergic reaction to a drug should refuse any switching in their drugs	257 (65.9%)	133 (34.1%)	
Total average knowledge scores out of 6			**3.67**

Participants were asked questions to measure their knowledge about generic switching. Data are presented as frequency (percentage) [N (%)]. *ACEIs, Angiotensin-converting enzyme inhibitors; ARBs, Angiotensin receptor blockers; BBs, Beta-blockers; CCBs, Calcium channels blockers.

When asked about the safety of generic switching among different pharmacological classes, the best knowledge was regarding pain killers, neurological drugs, and immunosuppressants with 76.9%, 75.6%, and 73.6% knowing the correct answers respectively. Adequate knowledge was lacking in categories of cardiovascular drugs (10.3%), antiplatelet and anticoagulants (10.3%), and inhalers (24.1%) Table 3.

Multivariate linear regression analysis showed that significant predictors of knowledge were gender, GPS (Good and poor GPA), and family income with *p*-values of 0.001, 0.013, 0.012, and 0.045, respectively. Female students had a higher knowledge score compared to their male peers. Students with “good” or “poor” GPAs had a lower knowledge score compared to those with “excellent” GPAs. Moreover, students that belong to families with incomes lower than the national average had a lower knowledge score, Table 4.

**TABLE 4:** Predictors of knowledge score among medical students, *N* = 390.

	Univariate linear regression			Multivariate linear regression		
	B	95% CI	*p*-value	B	95% CI	*p*-value
Gender						
Male^α^						
Female	0.350	0.142–0.557	0.001	0.361	0.141–0.581	0.001
Year of study						
Fourth-year students^α^						
Fifth-year students	0.227	−0.039–0.493	0.094	0.244	−0.019–0.507	0.069
Sixth-year students	0.102	−0.146–0.351	0.418	0.189	−0.061–0.439	0.139
GPA						
Excellent^α^						
Very good	−0.206	−0.483–0.070	0.143	−0.239	−0.516–0.037	0.089
Good	−0.335	−0.619–-0.051	0.021	−0.367	−0.655–-0.079	0.013
Fair	−0.094	−0.882–0.694	0.815	−0.237	−1.020–0.546	0.552
Poor	−2.215	−4.224–-0.207	0.031	−2.548	−4.525–-0.571	0.012
Family income is lower than the national average						
No^α^						
Yes	−0.203	−0.428–0.021	0.075	−0.227	−0.449–-0.005	0.045
Do you own health insurance?						
No^α^						
Yes	0.176	−0.085–0.438	0.186	0.180	−0.076–0.437	0.168
Do you use any medications that are not related to chronic medical conditions?						
No^α^						
Yes	0.070	−0.142–0.283	0.514	0.016	−0.207–0.239	0.886
Do you use any medications for the management of chronic medical conditions?						
No^α^						
Yes	0.258	−0.049–0.564	0.099	0.215	−0.091–0.520	0.168

a Univariate and multivariate linear regression were used to find correlations between demographic characteristics and knowledge scores. *p*-values less than 0.05 were considered statistically significant. α: Used as a reference.

### Participant’s attitude

**FIGURE 1:**
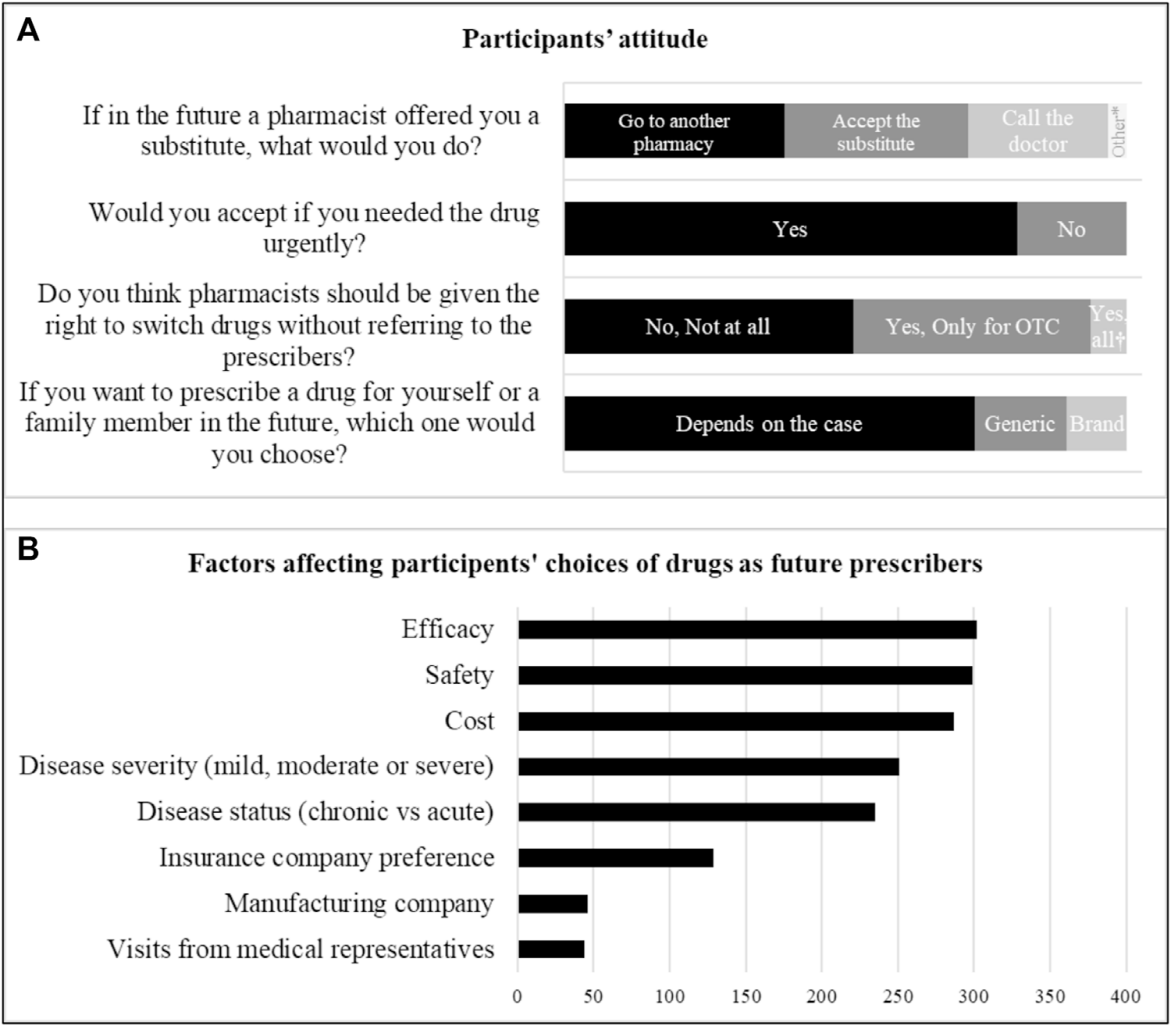
Participants’ attitude. Participants were asked about **(A)** their future actions as patients, their attitude towards pharmacists’ rights, their future choice of drugs as patients and **(B)** the factors affecting their choice of drugs as future prescribers (*N* = 390). *Participants stated that they can decide about the substitute offered by the pharmacists without referring to the doctor. ^†^Pharmacists should be given the right to do independent substitution on all drug classes.

Participants were asked questions about their future practices regarding generic switching, their drug choices as future patients and/or doctors, and their general opinions about expanding pharmacists’ rights to switch drugs. If in the future there was an unavailable drug in a prescription, 158 participants stated that they would go look in other pharmacies without accepting a substitution from the pharmacist or even calling the doctor, and 15 said that they would be able to judge the substitution offered by the pharmacist without referring to the prescriber (Figure 1A). However, most of the participants (80.5%) said that they would accept an alternative from the pharmacist if they needed the drug urgently (Figure 1A). Half of the participants (51.5%) believe that pharmacists should not be given the right to change any drug in the prescription (Figure 1A). Efficacy, safety, and cost were the major determinants of drug choice in participants’ future prescriptions while manufacturing companies and visits from medical representatives will have the least impact on their choices of drugs (Figure 1B).

When comparing attitude with knowledge, there was no correlation between knowledge score and the attitudes of the participants towards pharmacists’ right to drug switching (*p* = 0.166). Additionally, no association was found between the knowledge score and the attitudes of participants concerning accepting the switch by the pharmacist or referring to the physician (*p* = 0.444).

## Discussion

This is the first study in Jordan to study generic switching from the perspective of medical students. The results showed that in almost half the visits participants made to doctors’ clinics as patients, the doctors used specific drug names in the prescription - either the originator brand names or the name of generic substitutes. In only 6.9% of cases did doctors use the active ingredient names in their prescriptions. A similar study conducted in Tanzania, revealed that prescribing drugs using their brand names is a common practice (Mwita et al., 2022). The current study shows that, although illegal, independent generic switching is a common practice among community pharmacists in Jordan. In addition, it showed that when they were offered alternatives, only a quarter of participants chose the cheapest one. This differs to the results of a previous (2012) study of the general population in Jordan, where 93% of participants stated that they would choose the cheapest drug available if they had the option to do so (El-dahiyat and Kayyali, 2013a). In the current study, 30% of participants noticed that COVID-19 had affected drug availability in the local market. However, this was only a measure of participants’ personal experience, and since there are no published studies to confirm that there were indeed drug shortages in Jordan during COVID-19, the finding is considered inconclusive, and more studies would be needed to confirm it. There have, however, been other studies conducted internationally that showed COVID-19 to have resulted in drug shortages (Panic et al., 2020; Al Zoubi et al., 2021; Sample Size Calculator by Raosoft, 2022).

Unlike other studies, this study did not ask the students about the definitions of brand and generic drugs. Instead, definitions were provided before starting the questionnaire, and questions about students’ knowledge were more specific and detailed. Questions were designed to measure students’ knowledge about patients’ rights, pharmacists’ rights, the safety of generic switching, differences between branded and generic drugs, monitoring after drug substitution in chronic diseases, and generic switching in special populations. The average knowledge score was (3.67/6.00). The highest knowledge score was regarding patients’ rights followed by monitoring after generic switching in chronic diseases. Knowledge scores were lowest concerning the safety of generic switching in certain conditions and pharmacological classes, with a knowledge score of 0.41/1.00. This suggests that medical students, unfortunately, are not well informed about generic drugs and generic switching and this highlights the necessity of including this topic in pharmacology or therapeutic courses to improve students’ knowledge. When asked about the differences between brand and generic drugs in terms of efficacy, safety, and cost, 61.8%, 54.6%, and 66.2% gave the correct answers, respectively. Although knowledge can still be improved in these areas, the scores were higher than those obtained from medical students in other countries (Othman et al., 2016; Asif et al., 2017; Sharif et al., 2020). A similar study from Sierra Leone, that compared the knowledge of medical, pharmacy, and nursing students, showed different levels of knowledge among medical students compared to the current study. Compared to the medical students in Jordan, they showed better, similar, and lower levels of knowledge about efficacy, safety, and cost, respectively (James et al., 2018).

When analysing the data for predictors of knowledge, female students, superior academic achievement, and higher socioeconomic status were associated with better knowledge. In contrast, seniority, coverage with medical insurance, and the regular use of drugs (whether chronic or not) were not significantly related to participants’ knowledge. Only a few other studies have measured correlations between demographics and knowledge. However, a study in Yemen did not find gender to be a predictor of knowledge in final-year medical students, in contrast to the results of the current study (Othman et al., 2016). This conflicts with a similar study from Pakistan, however, which did find gender a predictor of knowledge, and also that the year of study was correlated with knowledge (Asif et al., 2017).

A third of the participants from this study stated that they trust pharmacists, and that they would find it acceptable for pharmacists to offer them a substitution drug in the future. A higher percentage of participants indicated that they would accept a substitution if they needed the drug urgently. This suggests that the urgency of patients’ need for drugs can affect their attitudes. Half (51.5%) of the medical students in the current study were against expanding the pharmacist’s role in generic switching to allow them to make substitutions independently (without referring to the prescribers). Medical students from other studies had a more positive attitude to this proposal, and were more open to it, as they see pharmacists as capable of giving helpful guidance and information about drugs (James et al., 2018; Sharif et al., 2020). This emphasises the importance of incorporating this topic into the curricula of Jordan’s medical students, and of highlighting the pharmacists’ key role in generic switching.

When asked about their future choices between generic or branded drugs as patients, almost three-quarters said that the choice would depend on the case, and only 16% said that they would choose generic drugs. This implies that the students have a low trust of generic drugs and is an issue that needs to be addressed. Medical students’ trust would be improved by increasing their knowledge about generic drugs, and the strict approval and registration processes they go through before becoming available in the market. A study conducted by Tobin and Laing, on medical students from Boston University, showed that students would only choose generic drugs for certain illnesses (Tobin and Laing, 2015). The current study, also, revealed that effectiveness, safety, and cost are the most important determinants of drug choice when participants were responding as future prescribers—followed by disease severity. In some studies participants have reported that visits from medical representatives, free drug samples, and promotions from pharmaceutical companies, would affect their choice of drugs (Tobin and Laing, 2015; James et al., 2018; Bookwalter, 2021). In the current study, however, only 44 participants (11.3%) said that such factors would influence their decisions. This indicates that the majority of participants would be willing to prescribe generic drugs to their patients in the future.

The originality of this study is its main strength. It is the first study, to the best of our knowledge, to examine each participant’s response as a patient, a student, and a future prescriber, concurrently. Additionally, this study intentionally used in-depth questions about generic switching in relation to current policies, pharmacological knowledge, differences between brand and generic drugs, monitoring after drug substitution in chronic diseases, and generic switching in special populations (Gregory, 1997, Chua et al., 2010; Gyawali et al., 2016).

## Conclusion

Sufficient knowledge about generic drugs, and generic switching, among medical students in Jordan is lacking. Students need to be more knowledgeable about the current policies and regulations of this practice, and the role of each healthcare worker it involves. Additionally, detailed information about the safety of drug substitution in generic switching should be introduced to pharmacology and/or therapeutic medical school courses. Students should also be encouraged to develop a positive attitude towards the pharmacist’s role in this practice. Another key finding of this study is that independent generic switching, although illegal, is a commonly practiced by community pharmacists in Jordan. This necessitates urgent intervention by the Ministry of Health and the Jordan Pharmacists Association. This should involve random inspections of pharmacies to limit unauthorised switching and avoid any medication errors or patient harm that may result from it. At the same time, pharmacists should be educated about their rights and limitations. However, due to the importance of generic switching in reducing healthcare expenditure, the authors point out that legalising the practice could be beneficial—as long as the pharmacists in Jordan received sufficient training and education. Such a step would also require that it was confined to a regularly updated safe exchange drug list published by the Ministry of Health.

## Data Availability

The original contributions presented in the study are included in the article/Supplementary Material, further inquiries can be directed to the corresponding author.
