# Supplementary material for: Generic switching: Do future physicians in Jordan have enough knowledge and a positive attitude?
Source: Front Pharmacol. 2022 Dec 6;13:1037112. doi: 10.3389/fphar.2022.1037112 (PMC9763699; doi:10.3389/fphar.2022.1037112)
Supplement: Supplementary file 1 [file Table1.DOCX]

**Supplementary Table 1: The questionnaire.**

| **Demographics** | | | | | | | | | | | | | | | | |
| --- | --- | --- | --- | --- | --- | --- | --- | --- | --- | --- | --- | --- | --- | --- | --- | --- |
| I am a student at | | BAU | JU | | | | | JUST | | | HU | | MU | | | YU |
| Gender | | Male | | | | | | | | | female | | | | | |
| Year of study | | 4th | | | | | | 5th | | | | | 6th | | | |
| GPA | | Excellent | | | Very good | | | | | Good | | Fair | | | Poor | |
| Number of Family members | | 3-4 | | | | | | 5-6 | | | | | 7 or more | | | |
| Family monthly income | | <1000 JOD | | | | | | 1000-1999 JOD | | | | | >2000 JOD | | | |
| Type of insurance | | Civil | | | | Private | | | | | Military | | | None | | |
| Do you suffer from any chronic disease that requires chronic treatment | | NO | | | | | | | | | Yes * | | | | | |
| * If yes: | What do you suffer from? (You can choose more than one) | - Asthma - Type 1 DM - MS | | | | | - SLE - Epilepsy - Depression | | | | | - Thyroid dysfunction - Allergies - Other: …. | | | | |
| Do you use any of the following medications that are not related to chronic diseases? You can choose more than one | | - Vitamins and minerals (e.g. Vit C, Vit D, Vit B12, Calcium, Magnesium ...) - Supplements (e.g. Fish oil, Probiotics, Herbal supplements....) - Collagen - Weight reduction drugs - Acne medications - Sleeping aids - Laxatives - Irritable bowel syndrome drugs - I don't use any drug that is not related to a chronic disease - Other (Please specify): ……………….. | | | | | | | | | | | | | | |
| **Experience** | | | | | | | | | | | | | | | | |
| From your past visits to doctors, do they write drugs in the prescription using brand/ generic names or using the active ingredient? | | Brand/ generic name | | | | Active  ingredients | | | | | I have seen both scenarios | | | I don’t  remember | | |
| Have you ever been using a brand drug then you had to use its substitute (generic drug) for any reason? | | Yes, it happened to me | | | | | | | | | No, never | | | | | |
| Have you ever asked the pharmacist for a substitute for a drug prescribed by your doctor? | | Yes | | | | | | | | | No | | | | | |
| Have you ever found one or more of the drugs in a prescription not available at the pharmacy? | | Yes, many times* | | | | | | Yes, it happened a few times* | | | | | No, it never happened | | | |
| * If yes: | What was the pharmacist's response to the unavailable drug? (You can choose more than one) |  They called the doctor to ask for a substitute   They informed me that there is a good substitute without referring to the doctor   They offered to order it for me   They asked me to look in another pharmacy | | | | | | | | | | | | | | |
| When you were offered multiple generic drugs, which one did you choose? (You can choose more than one) | | - The cheapest - The one you tried before - The most expensive - The one from companies you trust - The foreign drug - Other: ……….. | | | | | | | | | | | | | | |
| Have you noticed any change in drug availability due to COVID -19? | | Yes | | | | | | No | | | | | I don’t know | | | |
| **Knowledge** | | | | | | | | | | | | | | | | |
| Do patients have the right to ask for a substitute for a drug a doctor prescribed for them? | | Yes | | | | | | No | | | | | I don’t know | | | |
| Do pharmacists have the right to change a drug a doctor prescribed for a patient? | | Not at all | | Only if they refer to the prescriber | | | | | | | Yes, only for OTC drugs | | | Yes of course for all drugs | | |
| The following drugs can be switched safely: | | | | | | | | | | | | | | | | |
| CV drugs (ACEI, ARBs,BB,CCB) | | Agree | | | | | | I don’t know | | | | | Disagree | | | |
| Antiplatelet and anticoagulants | | Agree | | | | | | I don’t know | | | | | Disagree | | | |
| Inhalers | | Agree | | | | | | I don’t know | | | | | Disagree | | | |
| Immunosuppressants | | Agree | | | | | | I don’t know | | | | | Disagree | | | |
| Pain killers | | Agree | | | | | | I don’t know | | | | | Disagree | | | |
| Antibiotic | | Agree | | | | | | I don’t know | | | | | Disagree | | | |
| Hormones (levothyroxine) | | Agree | | | | | | I don’t know | | | | | Disagree | | | |
| Allergy drugs (antihistamines) | | Agree | | | | | | I don’t know | | | | | Disagree | | | |
| Neurological disorder drugs (Antiepileptics) | | Agree | | | | | | I don’t know | | | | | Disagree | | | |
| Contraceptives | | Agree | | | | | | I don’t know | | | | | Disagree | | | |
| Corticosteroids | | Agree | | | | | | I don’t know | | | | | Disagree | | | |
| Which one is more …… | | | | | | | | | | | | | | | | |
| Effective | | Brand drug | | | | | | Generic drugs | | | | | They are similar | | | |
| Safe | | Brand drug | | | | | | Generic drugs | | | | | They are similar | | | |
| Expensive | | Brand drug | | | | | | Generic drugs | | | | | They are similar | | | |
| Do generic switching in chronic diseases needs more frequent monitoring? | | Yes | | | | | | No | | | | | I don’t know | | | |
| Do people who ever experienced an allergic reaction to a drug should refuse any switching in their drugs? | | Yes | | | | | | No | | | | | I don’t know | | | |
| **Attitude** | | | | | | | | | | | | | | | | |
| If this happened to you in the future and the pharmacist offered a substitute, what will you do? | | - I trust the pharmacist and will accept the substitute - I will ask the pharmacist to call the doctor - I will go to another pharmacy - Other: … | | | | | | | | | | | | | | |
| Will you accept if you need the drug urgently | | Yes | | | | | | | | | No | | | | | |
| Do you think pharmacists should be given the right to switch drugs without referring to the prescribers? | | - Yes of course for all drugs - Only for OTC drugs - No at all | | | | | | | | | | | | | | |
| If you want to prescribe a drug for yourself or a family member in the future, which one will you choose? | | The brand drug | | | | Any generic drug | | | | | Generic drugs from certain companies | | | Depends on the case | | |
| As a doctor in the future, which one of the following will affect your choice of prescribed drugs? (You can choose more than one) | | - Efficacy - Safety - Cost - Disease status (chronic vs acute) | | | | | | | - Disease severity (mild, moderate, or severe) - Manufacturing company - Visits from medical representatives - Insurance company preference | | | | | | | |
